# Relationships between plasma leptin levels, *leptin* G2548A, *leptin receptor* Gln223Arg polymorphisms and gestational diabetes mellitus in Chinese population

**DOI:** 10.1038/srep23948

**Published:** 2016-04-01

**Authors:** Mei Yang, Songxu Peng, Wei Li, Zhihua Wan, Linlin Fan, Yukai Du

**Affiliations:** 1 Department of Maternal and Child Health, Tongji Medical College, Huazhong University of Science and Technology, Wuhan, China; 2 Department of Pharmacology, Tongji Medical College, Huazhong University of Science and Technology, Wuhan, China

## Abstract

The purposes of this study were to examine concentrations of leptin and biochemical parameters in gestational diabetes mellitus (GDM) patients and normal glucose tolerance (NGT) individuals, and also to explore the links of *leptin* (*LEP*) G2548A and *leptin receptor* (*LEPR*) Gln223Arg polymorphisms with leptin levels and GDM risk among Chinese. Our study included 357 GDM and 355 NGT individuals who were at 24~30 gestational weeks. Plasma leptin and insulin levels were analyzed by ELISA. Gene polymorphisms were genotyped using TaqMan real-time polymerase chain reaction assay. The results showed that plasma leptin levels were significantly higher in the impaired fasting glucose (IFG) group than NGT group (34.35 (26.54, 56.48) ng/mL vs 26.31 (17.99, 37.87) ng/mL, *P* < 0.05). Plasma leptin levels correlated with plasma fasting insulin levels, pre-pregnant body mass index, homeostasis model assessment-insulin resistance and quantitative insulin sensitivity check index both in GDM and NGT group (*P* < 0.05). However, neither *LEP* G2548A nor *LEPR* Gln223Arg polymorphisms were significantly associated with GDM risk and plasma leptin levels (*P* > 0.05). Our findings showed that high leptin level was associated with GDM. And larger and more rigorous researches were needed to further explore the association of *LEP* and *LEPR* gene polymorphisms and GDM among Chinese population.

Gestational diabetes mellitus (GDM) is characterized by any degree of glucose intolerance with onset or first recognition during pregnancy^1^. Due to lifestyle changes, more and more pregnant women develop GDM. In Chinese population, a longitudinal study reported that during October 2010 to August 2012, 7.5% pregnant women were diagnosed with GDM upon the criteria of International Association of Diabetes and Pregnancy Study Groups (IADPSG)^2^. The adverse outcomes associated with GDM for both the mother and the offspring are diverse. They include short-term complications such as macrosomia, neonatal hypoglycemia, preeclampsia, as well as long-term risk of type 2 diabetes mellitus, obesity and cardiovascular diseases both in the mother and in their offspring^3^,^4^. As a prediabetic status, GDM is thought to be associated with hyperinsulinemia and insulin resistance in general, but the pathophysiology of GDM has not been thoroughly investigated.

Leptin, a polypeptide hormone, binds to the hypothalamic leptin receptor to decrease food intake and increase energy expenditure^5^. Nowadays, emerging evidences suggest that leptin receptors also distribute in peripheral region. In addition, leptin has a direct effect on insulin sensitivity as well as insulin secretion^6^,^7^,^8^,^9^. Except for adipocytes, leptin can also be produced by non-adipose tissues such as stomach, intestine, and in particular the placenta in humans. In pregnant women, because of the increased fat mass and the presence of placenta, maternal leptin concentrations increase 2 to 3-fold above non-pregnant concentration, with the peak occurring around 28 weeks of gestation^10^.

Concerning the role of leptin in maternal metabolism and maternal glucose homeostasis regulation, an analysis of plasma leptin levels and GDM is of considerable interest. There are some reports regarding the levels of maternal leptin in GDM, though the results are conflicting. It has been reported that the levels of leptin were increased^11^,^12^,^13^, decreased^14^ or unchanged^15^,^16^,^17^ in GDM. Noureldeen, A F *et al.* reported that plasma leptin levels did not significantly change at 2^nd^ trimester but decrease at 3^rd^ trimester among GDM women^18^. Result from a cohort study showed that each 10 ng/ml increase in the leptin concentration in early pregnancy was associated with a 20% increase in GDM risk (RR = 1.2, 95% CI: 1.0–1.3)^19^.

The *leptin* (*LEP*) G2548A polymorphism has been associated with increased leptin production and plasma secretion from adipocytes^20^. Meanwhile, mutation in *leptin receptor* (*LEPR*) Gln223Arg results in impaired signaling capacity of leptin receptor^21^. The relationships between these two gene polymorphisms and obesity or type 2 diabetes are widely studied^21^,^22^,^23^. But few studies researched the links with GDM^24^. What’s more, the relationship of *LEP* G2548A and *LEPR* Gln223Arg with GDM among Chinese has not been investigated to date.

So the aim of our study was to measure the plasma leptin levels in pregnant women with and without GDM, as well as investigate the frequency occurrence of *LEP* G2548A and *LEPR* Gln223Arg polymorphisms in Chinese population. Moreover, the relationships between metabolic parameters describing glycemic control and these two single nucleotide polymorphisms (SNPs) were evaluated.

## Results

### Baseline characteristics

The clinical characteristics of GDM and normal glucose tolerance (NGT) groups were summarized in Table 1. Both groups were comparable for gestational age (*t* = 1.30, *P* = 0.19). Compared with the NGT individuals, the pregnant women with GDM had a higher age, pre-pregnant body mass index (BMI), BMI during 24~30 gestational weeks, systolic blood pressure, diastolic blood pressure and fasting blood glucose (*P* < 0.001). Furthermore, as presented in Table 2, the women with impaired fasting glucose (IFG) had significantly higher fasting plasma leptin levels than NGT group (IFG vs NGT: 34.35 (26.54, 56.48) ng/mL vs 26.31 (17.99, 37.87) ng/mL, *P* < 0.05). There were significant difference in fasting plasma insulin levels between IFG or impaired fasting and stimulated plasma glucose (IFSG) and NGT group (9.53 (7.66–11.47) μU/mL or 10.46 (7.877–13.14) μU/mL vs 6.87 (5.51–8.42) μU/mL, *P* < 0.001). Insulin resistance assessed by homeostasis model assessment-insulin resistance (HOMA-IR) was significantly higher in IFG and IFSG patients than NGT individuals. In contrast, insulin sensitively calculated from quantitative insulin sensitivity check index (QUICKI) was significantly lower in IFG and IFSG patients. However, no significantly differences between IGT and NGT groups in these four biochemical variables were found (*P* > 0.05).

### Leptin and other variables

Plasma leptin level was positively correlated with pre-pregnant BMI (*r*_*s*_ = 0.45, *P* < 0.001), systolic blood pressure (*r*_*s*_ = 0.16, *P* < 0.001), diastolic blood pressure (*r*_*s*_ = 0.08, *P* = 0.03), fasting insulin (*r*_*s*_ = 0.45, *P* < 0.001), fasting plasma glucose (*r*_*s*_ = 0.23, *P* < 0.001) and HOMA-IR (*r*_*s*_ = 0.44, *P* < 0.001), but negatively correlated with QUICKI (*r*_*s*_ = −0.44, *P* < 0.001) when took all subjects into account. Furthermore, the Spearman’s rank coefficients between leptin levels and fasting insulin (*r*_*s*_ = 0.53, *P* < 0.001), fasting plasma glucose (*r*_*s*_ = 0.24, *P* < 0.001) and HOMA-IR (*r*_*s*_ = 0.51, *P* < 0.001), QUICKI (*r*_*s*_ = −0.51, *P* < 0.001) were mildly increased in GDM group, but reduced in NGT group (data not shown).

### Characteristics of the study population according to *LEP* G2548A and *LEPR* Gln223Arg genotypes

An analysis of metabolic characteristics across genotypes was performed (Table 3). There was significantly difference between *LEP* G2548A polymorphism and BMI (*F* = 4.48, *P* = 0.01). And the 2548GA genotype had significantly higher BMI than 2548GG (21.17 ± 3.05 kg/m^2^ vs 20.15 ± 2.54 kg/m^2^), even though the BMI between 2548AA and 2548GG genotype was similar. But the differences between fasting leptin, fasting insulin, HOMA-IR, QUICKI among *LEP* G2548A or *LEPR* Gln223Arg genotypes were not significant in our study (*P* > 0.05).

### Association between *LEP* G2548A and *LEPR* Gln223Arg polymorphisms and GDM risk

The genotype distributions for the *LEP* G2548A and *LEPR* Gln223Arg showed no deviation from Hardy-Weinberg equilibrium both in cases and controls (*P* > 0.05). The 2548G allelic frequencies were 0.290 and 0.250 in GDM and NGT pregnant women respectively. And 223Arg allelic frequencies were 0.111 and 0.108 in GDM and NGT pregnant women respectively. As presented in Table 4, no significant differences in genotype distribution and recessive model of the *LEP* G2548A and *LEPR* Gln223Arg had been found between GDM and NGT subjects after adjusted for age and BMI (*P* > 0.05). Neither in dominant and co-dominant model (data not shown). We did not find a trend for association between *LEP* G2548A and *LEPR* Gln223Arg and GDM (*P trend* > 0.05). The interactions of the two polymorphisms also showed insignificant relationships with GDM risk (data not shown).

## Discussion

To our knowledge, this was the first study examining the links of leptin level, *LEP* G2548A and *LEPR* Gln223Arg polymorphisms in GDM patients among Chinese. The key results of this study including that leptin levels were significantly higher in IFG group, but neither of the two polymorphisms showed significant links to leptin levels and GDM risk.

In 2010, the IADPSG issued new guidelines for the diagnosis and classification of hyperglycemia in pregnancy. According to those recommendations, a diagnosis of GDM referred to any degree of glucose intolerance with high heterogeneity. In our study, GDM patients were divided into 3 groups to compare the metabolic parameters with NGT group. Compared with the NGT group, higher leptin levels were found in the IFG group, consistent with the previous study^19^,^25^. Similarly, significantly higher fasting insulin levels, HOMO-IR and lower QUICKI were also noted in IFG and IFSG group, namely impaired fasting glucose individuals, than NGT group, but not in IGT group. Maybe there were different pathogenesis between impaired fasting glucose individuals and impaired glucose tolerance patients, further studies were needed to reveal the mechanism.

In the current study, we used HOMA-IR and QUICKI for insulin resistance and sensitivity. As predicted, the positive and negative correlations were found between plasma leptin levels and HOMA-IR and QUICKI respectively. These correlations were confirmed in some studies^26^,^27^, but not all^28^. The observed positive correlations between plasma leptin concentrations and the maternal pre-pregnant BMI was in accordance with many previous studies both in GDM group and NGT group^11^,^14^. But in Mokhtari *et al.*’s study, the relationship existed merely in GDM group^16^.

For *LEP* G2548A polymorphism, the frequency of G allele was 26.98% in our study similar with the results from Taiwanese^29^, but different from other populations where the G allele frequency was higher than the A allele^21^,^24^,^30^,^31^. Few researches explored the association of *LEP* G2548A polymorphism and GDM. A Czech study with 48 GDM and 53 controls showed that AA and AG genotype carries had a significantly higher risk for GDM against those carrying GG genotype^24^. However, this association were not duplicated in our population. For *LEPR* Gln223Arg polymorphism, genotype and allele frequencies observed in our study were similar to those described in other populations^30^. But we failed to find a significant relationship in Chinese population. In our study, we did not find a significant trend for association between these two polymorphisms and GDM, which might be mediated through the association between BMI. To reveal the real trend, another rigorous study with BMI-matched cases and controls should be done.

With the minor allele frequencies (MAF) of *LEP* G2548A (0.250) and *LEPR* Gln223Arg (0.108) in controls, sample size (357 patients and 355 controls), expected OR (1.50), we calculated the power of the study. The power of this study to detect the association for *LEP* G2548A and *LEPR* Gln223Arg and GDM were 0.69 and 0.44 respectively. With the limited statistic power, it should be cautious to reject the association between these two polymorphisms and GDM. A much larger sample size study showed be conducted in further analysis.

Moreover, the relationships between metabolic parameters and these two polymorphisms were evaluated. Though previous study reported *LEP* G2548A polymorphism had a strong influence on leptin gene expression^32^, data in Melanesian^33^, Thai^34^ and our Chinese subjects found no relationship between *LEP* G2548A gene polymorphism and leptin levels, inconsistent with the results in Egyptian^35^ and Romanian subjects^23^. With regard to relationships between *LEPR* Gln223Arg gene polymorphism and leptin levels, a relationship was found in Thai^34^, Dutch^36^ and Romanian subjects^23^, but neither in Turkish children^37^ nor in our Chinese subjects. Our results also showed no association between the *LEPR* Gln223Arg polymorphism and other metabolic parameters in agreement with the conclusion of Heo *et al.*’s meta-analysis^38^. But other studies suggested that *LEPR* Gln223Arg polymorphism was associated with higher insulin resistance and BMI^30^.

These inconsistencies among the results might arise from (1) the different genetic backgrounds or environmental conditions of the populations studied; (2) sample size of the population; (3) the different diagnosis criteria of GDM patients: with the criteria of IADPSG more pregnant women were diagnosed with GDM, which meant that part of GDM patients in our study were considered as controls in other studies, and this would virtually influence the results; (4) different target population and different timing of maternal blood collection: many previous studies were conducted in non-pregnant women, but the regulation of leptin might be very different during pregnancy. Even during pregnant, different trimester showed different metabolic characteristics of leptin.

Even with a larger sample size, our study had some limitations. First was the absence of the measurement of fat mass and body fat distribution besides BMI. Second, the patients were enrolled after the diagnosis of GDM and we could not determine whether any observed alterations in plasma leptin levels preceded GDM. Third, even though the age and BMI were adjusted for analysis, the confounding factors could not be excluded completely.

In summary, the present study revealed a mildly increase in fasting plasma leptin level in IFG patients than NGT individuals, as well as a significantly higher plasma insulin, higher HOMA-IR values and lower QUICKI values in IFG and IFSG group than NGT group. Furthermore, the fasting plasma leptin levels were positive correlated with fasting plasma insulin, HOMA-IR, but negative correlated with QUICKI. However, no significant associations of the *LEP* G2548A and *LEPR* Gln223Arg polymorphisms with GDM risk and with leptin level were found in Chinese population.

## Methods

### Study Population

A total of 712 (357 GDM and 355 NGT) pregnant women were included in our study. GDM patients were consecutively recruited between January 15 to September 31, 2015 at Maternal and Child Health Care Hospital of Bao’an District, Shenzhen, China. NGT individuals were randomly selected at the same outpatient during the same time. All subjects were enrolled during 24 and 30 gestational weeks right after the 75 g oral glucose tolerance test (OGTT). The diagnosis of GDM was based upon the criteria of IADPSG by which one or more of the following plasma values from 75 g OGTT must be equaled or exceeded: fasting 5.1 mmol/L, 1 h 10.0 mmol/L and 2 h 8.5 mmol/L^39^. Pregnant women who reached these thresholds were included in GDM group, otherwise in NGT group. As for GDM group, women were further categorized as: (1) IFG if the fasting plasma glucose (FPG) was ≥5.1 mmol/L but with normal 1 h and 2 h OGTT values; (2) IGT: if FPG was <5.1 mmol/L but with elevated 1 h or 2 h OGTT values; (3) IFSG: if both FPG and 1 h or 2 h OGTT values were elevated. Our study only included women with only diet-treated GDM. Exclusion criteria were: age <18 years; pre-gestational diabetes (type 1 or 2); multiple pregnancy; complicated pregnancy; chronic disease or any other major medical condition that might affect glucose or leptin regulation. All subjects were unrelated Han Chinese.

At recruitment, maternal blood samples in the fasted state (8 to 12 h fast, no more than 12 h) were collected by interviewers. The physical examination included an assessment of height, weight and blood pressure was measured by standard protocol and BMI was calculated by dividing the weight in kilograms by the height in meters squared. Pre-pregnancy weight was obtained by history. The study was approved by the institutional review boards of Tongji Medical College, Huazhong University of Science & Technology, and all subjects provided informed consent for participation. The methods were carried out in accordance with the principles of the Declaration of Helsinki.

### Laboratory measurements

2 ml blood from the antecubital vein were immediately placed on ice and separated into plasma and cells within 30 min, then distributed in aliquots and stored at −80 °C until analysis. Genomic DNA was isolated from 0.5 ml blood cells using the approved guideline of the Relax Gene Blood DNA System DP348 (Tiangen, China). *LEP* G2548A (rs7799039) and *LEPR* Gln200Arg (rs1137101) polymorphisms were genotyped using the TaqMan real-time polymerase chain reaction (PCR) assay (Applied Biosystems, CA) without knowledge of the case or control status of the subjects. The ABI Prism 7900HT Sequence Detection System was applied to read the reacted plates and to analyze the endpoint fluorescence. In our study, the call rate for *LEP* G2548A and *LEPR* Gln200Arg polymorphisms were 97.61% and 99.02%.

Fasting plasma glucose measurements were performed by hexokinase method on the Cobas 8000 Modular Analyzer Series (Roche, Mannheim). Fasting plasma insulin was determined by enzyme-linked immunosorbent assay (ELISA) using the Insulin ELISA 10-1113-10 kit (Mercodia, Sweden), as were also plasma levels of leptin (4Abiotech, China). The absorbance was measured at 450 nm using the Automatic Microplate Reader (Syngene, USA). HOMA-IR and QUICKI were applied to assess insulin resistance and sensitivity, respectively. HOMA-IR was calculated as: fasting plasma glucose (mmol/L) × fasting plasma insulin (μU/mL)/22.5. QUICKI is defined as: 1/(logI0 + logG0), where I0 was fasting plasma insulin level (μU/mL) and G0 was fasting plasma glucose level (mg/dL)^40^.

### Statistical analysis

Normality of distribution for continuous variables was tested by the Kolmogorov-Smirnov test. Normal distribution data were presented as mean ± SD, and the differences among groups were compared by unpaired Student’s *t*-test or analysis of variance (ANOVA). For non-normal distribution data, median (25^th^–75^th^ interquartile range) was applied, and Mann-Whitney U or Kruskal-Wallis test were performed for comparison among groups. The multiple comparison was conducted by Dunnett-t test. The difference in genotype distribution as well as consistency of genotype distribution with Hardy-Weinberg equilibrium were tested using the chi-square test. Spearman rank correlation was used to calculated correlations between plasma leptin level and other biochemical variables. Logistic regression was preformed to evaluate the association of the genotypes and GDM risk. *P* < 0.05 was accepted as statistically significant. Cochran-Armitage trend test was used to calculate the gene-dose effect. Analyses were performed with SPSS Software, Version 18.0 for Windows (SPSS Inc., Chicago, IL, USA).

## Additional Information

**How to cite this article**: Yang, M. *et al.* Relationships between plasma leptin levels, *leptin* G2548A, *leptin receptor* Gln223Arg polymorphisms and gestational diabetes mellitus in Chinese population. *Sci. Rep.* **6**, 23948; doi: 10.1038/srep23948 (2016).

## Figures and Tables

**Table 1:** Clinical characteristics of the study population.

Variables	GDM group (n = 357)	NGT group (n = 355)	*P*
Age (years)	30.27 ± 4.45	27.89 ± 3.93	<0.001
Gestational age (weeks)	25.96 ± 1.31	26.10 ± 1.51	0.19
Pre-pregnant BMI (kg/m^2^)	21.33 ± 3.06	20.26 ± 2.42	<0.001
BMI during 24~30 gestational weeks (kg/m^2^)	24.50 ± 3.16	23.48 ± 2.70	<0.001
Systolic blood pressure (mmHg)	112.7 ± 12.32	108.9 ± 10.94	<0.001
Diastolic blood pressure (mmHg)	64.96 ± 7.96	62.74 ± 7.93	<0.001
Fasting plasma glucose (mmol/L)	4.67 ± 0.55	4.31 ± 0.29	<0.001

GDM: gestational diabetes mellitus; NGT: normal glucose tolerance; BMI: body mass index.

^†^*P* was calculated using unpaired Student’s *t*-test.

**Table 2:** Biochemical characteristics of the study population.

	IFG (n = 23)	IGT (n = 277)	IFSG (n = 57)	NGT (n = 355)	*P*
Fasting leptin (ng/mL)	34.35* (26.54–56.48)	25.86 (16.24–38.24)	31.30 (21.07–49.43)	26.31 (17.99–37.87)	0.005
Fasting insulin (μU/mL)	9.53* (7.66–11.47)	7.43 (5.66–9.50)	10.46* (7.87–13.14)	6.87 (5.51–8.42)	<0.001
HOMA-IR	2.18* (1.75–2.62)	1.49 (1.08–1.89)	2.52* (1.95–3.33)	1.30 (1.02–1.63)	<0.001
QUICKI	0.26* (0.25–0.27)	0.28 (0.27–0.31)	0.25* (0.23–0.26)	0.30 (0.28–0.32)	<0.001

IFG: impaired fasting glucose; IGT: impaired glucose tolerant; IFSG: impaired fasting and stimulated plasma glucose; NGT: normal glucose tolerance; HOMA-IR: homeostasis model assessment-insulin resistance; QUICKI: quantitative insulin sensitivity check index.

^†^*P* was calculated using Kruskal-Wallis test and the multiple comparison was conducted by Dunnett-t test.

^*^The difference was significant when compared with NGT group.

**Table 3:** Metabolic characteristics of the study population according to *LEP* G2548A and *LEPR* Gln233Arg genotypes.

	*LEP*G2548A				*LEPR*Gln233Arg			
	GG	GA	AA	*P*	Gln/ Gln	Arg/Gln	Arg/Arg	*P*
BMI (Kg/m^2^)	20.15 ± 2.54	21.17 ± 3.05	20.59 ± 2.65	0.01	20.79 ± 2.85	20.74 ± 2.60	20.81 ± 1.79	0.98
Fasting leptin (ng/ml)	24.95 (15.93–42.79)	28.47 (17.7–41.10)	25.02 (17.63–37.58)	0.26	27.65 (18.08–38.89)	25.83 (16.18–38.29)	20.17 (13.98–26.33)	0.27
Fasting insulin (μU/mL)	7.68 (6.47–8.92)	7.37 (5.74–9.44)	7.19 (5.61–9.16)	0.44	7.37 (5.64–9.20)	7.00 (5.81–9.42)	7.44 (6.10–9.81)	0.84
HOMA-IR	1.47 (1.24–1.94)	1.46 (1.09–1.98)	1.42 (1.08–1.89)	0.37	1.47 (1.08–1.92)	1.40 (1.11–1.87)	1.48 (1.14–2.14)	0.80
QUICKI	0.29 (0.26–0.30)	0.28 (0.26–0.31)	0.29 (0.27–0.31)	0.37	0.29 (0.27–0.31)	0.29 (0.27–0.31)	0.29 (0.26–0.31)	0.80

^†^*P* was calculated using ANOVA for normal distribution data and Kruskal-Wallis test for non-normal distribution data.

**Table 4:** Association between *LEP* G2548A and *LEPR* Gln233Arg polymorphisms and gestational diabetes mellitus.

	GDM group n (%)	NGT group n (%)	OR (95% CI)	*P*	*P trend*
*LEP* G2548A					
Allele					
A	493 (71.04)	522 (75.00)	1	0.10	
G	201 (28.96)	174 (25.00)	1.22 (0.96–1.55)		
Genotype					0.09
AA	172 (49.57)	195 (56.03)	1		
AG	149 (42.94)	132 (37.93)	1.20 (0.86–1.66)	0.28	
GG	26 (7.49)	21 (6.03)	1.41 (0.74–2.66)	0.30	
AG + GG	175 (50.43)	153 (43.96)	1.23 (0.90–1.68)	0.20	
*LEPR* Gln233Arg					
Allele					
Gln	628 (88.95)	628 (89.20)	1	0.88	
Arg	78 (11.05)	76 (10.80)	1.03 (0.73–1.43)		
Genotype					0.15
Gln/Gln	280 (79.32)	277 (78.69)	1		
Arg/Gln	68 (19.26)	74 (21.02)	0.91 (0.62–1.34)	0.64	
Arg/Arg	5 (1.42)	1 (0.28)	5.21 (0.58–46.93)	0.14	
Arg/Gln + Arg/Arg	73 (20.68)	75 (21.30)	0.97 (0.66–1.42)	0.87	

^†^ORs and 95% CIs were calculated by logistic regression after adjusting for age and BMI. *P* trends were calculated by Cochran-Armitage test.
